# Biomechanical causes for failure of the Physiomesh/Securestrap system

**DOI:** 10.1038/s41598-023-44940-8

**Published:** 2023-10-16

**Authors:** Mateusz Zamkowski, Agnieszka Tomaszewska, Izabela Lubowiecka, Maciej Śmietański

**Affiliations:** 1 Department of General Surgery and Hernia Center, Swissmed Hospital, Wileńska 44, 80-215 Gdańsk, Poland; 2 Department of Structural Mechanics, Faculty of Civil and Environmental Engineering, Gdańsk University of Technology, Gdańsk, Poland; 3 II Department of Radiology, Medical University of Gdańsk, Gdańsk, Poland

**Keywords:** Structural materials, Techniques and instrumentation, Medical research, Materials science, Quality of life

## Abstract

This study investigates the mechanical behavior of the Physiomesh/Securestrap system, a hernia repair system used for IPOM procedures associated with high failure rates. The study involved conducting mechanical experiments and numerical simulations to investigate the mechanical behavior of the Physiomesh/Securestrap system under pressure load. Uniaxial tension tests were conducted to determine the elasticity modulus of the Physiomesh in various directions and the strength of the mesh-tissue-staple junction. Ex-vivo experiments on porcine abdominal wall models were performed to observe the system's behavior under simulated intra-abdominal pressure load. Numerical simulations using finite element analysis were employed to support the experimental findings. The results reveal nonlinearity, anisotropy, and non-homogeneity in the mechanical properties of the Physiomesh, with stress concentration observed in the polydioxanone (PDO) stripe. The mesh-tissue junction exhibited inadequate fixation strength, leading to staple pull-out or breakage. The ex-vivo models demonstrated failure under higher pressure loads. Numerical simulations supported these findings, revealing the reaction forces exceeding the experimentally determined strength of the mesh-tissue-staple junction. The implications of this study extend beyond the specific case of the Physiomesh/Securestrap system, providing insights into the mechanics of implant-tissue systems. By considering biomechanical factors, researchers and clinicians can make informed decisions to develop improved implants that mimic the mechanics of a healthy abdominal wall. This knowledge can contribute to better surgical outcomes and reduce complications in abdominal hernia repair and to avoid similar failures in future.

## Introduction

Laparoendoscopic surgery and minimally invasive techniques are continually increasing their importance in treatment of abdominal hernias due to their association with a lower risk of perioperative infection, reduced risk of wound healing complications, and shorter hospital stay and recovery time^1^.

Surgeons operating on anterior abdominal wall defects have at their disposal several potential spaces to insert the implant. The mesh can be placed between the fascia and the subcutaneous tissue (onlay), under the rectus abdominis muscles (retrorectus), between the fascia and the peritoneum (underlay), and directly in the peritoneal cavity (IPOM—IntraPeritoneal Onlay Mesh)^2^. Each variant of implant location will differ in terms of both the forces acting on the implant, the healing and recovery process, and the risk of recurrence.

The original description of laparoscopic treatment of abdominal hernias concerned mainly the IPOM method^3,4^. IPOM requires placing the implant directly into the peritoneal cavity, to cover the abdominal wall defect with an appropriate margin, and fixing it with special joints (tackers, staples). That is connected with exposing the abdominal organs to direct contact with the mesh. Main concern of this way is a possible organ-mesh adhesion, which in turn may disturb the intestinal passage, and in critical situations even lead to fully symptomatic obstruction^5^. Initial optimism related to the introduction of IPOM was constantly reduced due to long-term post-operative complications. Currently, IPOM has its place in surgery of the anterior abdominal wall, but the indications have been severely limited and confined.

In the time of development of minimally invasive techniques and robotic techniques, the possibilities of implant placement have expanded^6^. The literature describes further innovative solutions, including eMILOS (endoscopic transhernial minimal invasive sublay mesh repair) and eTEP (extended totally extraperitoneal repair), which aim to avoid the need to implant the mesh directly into the peritoneal cavity^7–9^. This does not detract from the fact that IPOM is still the most frequently performed minimally invasive technique in terms of abdominal hernias.

Implant manufacturers focus on continuous development and improvement of their products by creating next generations of composite meshes to reduce the risk of adhesions. As yet, no ideal implant for use inside the peritoneal cavity has been found. The assumptions for an ideal mesh include withstanding forces induced by intraperitoneal pressure and body movement until the mesh is overgrown with the tissue, adapting to the shape of the abdominal cavity taking into account its specific structure, stimulating the body to overgrow the mesh with its own tissues, not generating adhesions with organs located inside the peritoneal cavity, and not leading to intra-abdominal injuries. In fact, the whole biomechanical system composed of abdominal tissues, mesh and staples must create a mechanically reliable system, which would mimic mechanics of healthy abdominal wall^10,11^. With this in mind, medical companies produce next generation of implants and fixation materials to find the golden standard. There has been a lot of expectations for covering the implant with an absorbable, non-adhesive barrier resorbed within 30–240 days after treatment. Examples of such meshes include Parietex Composite (Medtronic, Dublin, Ireland), Sepramesh IP Composite (BD, New Jersey, USA), Ventrio ST (BD, New Jersey, USA), and Ventralight ST (BD, New Jersey, USA). Fixation mechanisms also include a whole range of non-absorbable and absorbable materials.

New generations of meshes and modifications of the procedures, including closing the cavities before inserting the implant (so-called IPOM plus), robotic procedures, changing the staple arrangement scheme (single crown vs double crown technique) are intended to improve surgical outcome. However, not every novelty translates into improved clinical outcome^6,12^. The history of IPOM also involves spectacular failures that required stopping and re-evaluating the direction of development.

One example is implementation of the Physiomesh (PH) implant with a dedicated Securestrap fixation device by Ethicon (New Jersey, USA) in 2014. This supposedly improved generation of mesh, pushed the company out of the market and resulted in the need to pay out high, often multi-million, compensation^13,14^. In a randomized clinical trial (RCT) conducted in 2015, Pawlak et al. compared Physiomesh/Securestrap with Ventralight ST/Sorbafix systems in terms of pain, recurrence, and perioperative complications in laparoscopic IPOM procedures^15^. The trial was stopped for safety reasons, resulting from the high recurrence rate (20%) in the group of patients operated on with the Physiomesh/Securestrap system. In addition, patients in this group complained of much greater pain compared to patients operated with Ventralight ST/Sorbafix. Data from the German Herniamed Registry and Danish Hernia Database coincided with the results presented in the above RCT^16^.

The biological reasons for failure of the Physiomesh/Securestrap system were partly explained in animal models where, similarly to the aforementioned RTC, it was compared with a Ventralight ST implant and a dedicated Sorbafix fixation system^17^. A decisive advantage of Ventralight ST/Sorbafix was the lower percentage of inflammation, fibrosis, bleeding and angiogenesis at the implantation site, and a much greater overgrowth with host tissues in the 14-day period after implantation. However, no difference was observed in terms of adhesions, collagen deposition, necrosis generation, and mesh shrinkage percentage^17^. Alone, biological factors did not fully justify such a spectacular failure of Physiomesh/Securestrap system. This suggests that, apart from biological components, failure of Physiomesh/Securestrap is also due to biomechanical factors. Given this hypothesis, we investigated the mechanical behavior of the implant itself and the mesh-staple-tissue system ex-vivo. The objective was to establish the potential causes of such a high recurrence rate and pain. Our philosophy is that drawing conclusions from the failure of the Physiomesh/Securestrap system will not only help to avoid similar situations in the future, but it will also provide a better understanding of the mechanics of implant fixed to the human anterior abdominal wall.

## Materials and methods

To understand the cause of the very high rate of failures related to the use of the Physiomesh/Securestrap system in patients undergoing anterior abdominal wall reconstruction, we conducted mechanical experiments as well as numerical simulations of the system behaviour under physiological pressure load.

Three kinds of experiments were performed of Physiomesh. The elasticity of the implant was determined based on the uniaxial tension tests. The strength of the mesh-tissue junction made by the staple was identified in the uniaxial tests. Finally, ex-vivo experiments on the Physiomesh/Securestrap implanted to porcine abdominal wall were performed. Within the experiments the behaviour of that system under simulated ‘intra-abdominal’ pressure load was observed. All that allowed us to gain knowledge on the mechanical performance of the Physiomesh/Securestrap system and delivered data for numerical simulations of the system behaviour, complementing the biomechanical analysis.

### Materials used in the study

**Figure 1:**
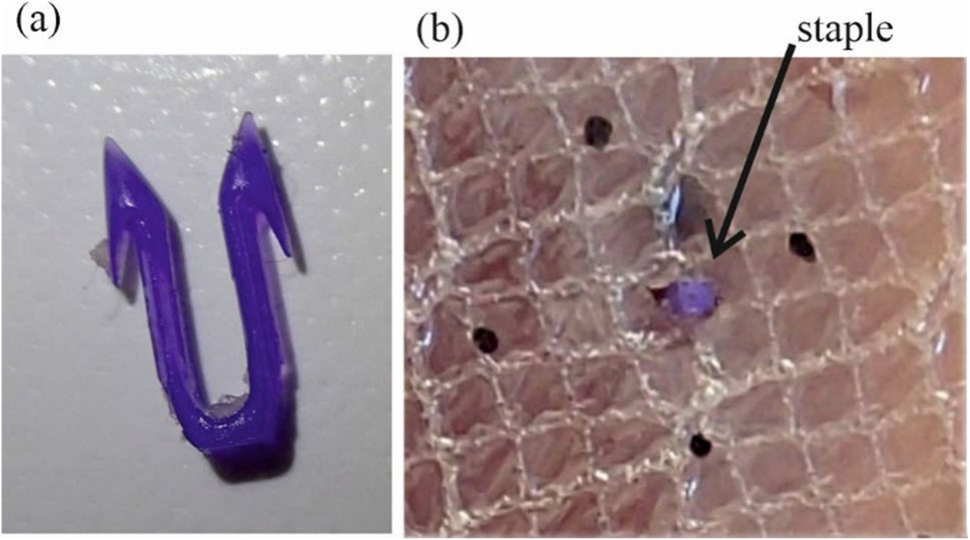
(**a**) Securestrap staple. (**b**) Physiomesh/ Securestrap system placed in a porcine tissue.

The study involved the Physiomesh implant made from monofilament polypropylene surrounded by polydioxanone (PDO) on both sides in order to maintain the bond with two poliglecaprone-25 absorbable barrier layers on both sides of the implant. Their purpose is to minimize adhesions between the mesh and the abdominal organs. In principle, the barrier should dissolve in vivo a few weeks after implantation. A violet stripe made of PDO is inserted through the center of the implant on one side, as an orientation marker. According to the manufacturer recommendation the mesh should be oriented in the abdominal wall so that the stripe aligns the cranio-caudal direction of the body^17^. Physiomesh was available in a kit with Securestrap staples, which allow fixation of the implant to the anterior abdominal wall in the IPOM Procedure. Securestrap staple’s substance is a mixture of PDO and lactide copolymer, which should be completely resorbed in vivo 12 months after implantation. Its shape resembles very narrow ‘U’ letter with two “teeth” of 6.7 mm length each, which provide two sites of fixation^18^. Both materials are shown in Fig. 1.

### Mesh-staples-tissue model behaviour under ‘intra-abdominal’ pressure load

Ex-vivo experiments on the physical models of hernia operated with a mesh and staples of interest allowed to deduce on such system behavior in the human body. The authors used a pressure chamber, which was described and validated in earlier studies^19–21^. The device allows simulating a static or dynamic action of an ‘intra-abdominal’ pressure on a sample placed inside. Three similar models were built of a side part of the porcine abdominal wall, in which hernia orifice (diameter of 5 cm) was cut. The hernia was ‘operated’ with the use of Physiomesh attached by Securestrap staples to the fascia. Twelve staples were used in each model, evenly spaced in a single crown layout. The crown had a diameter of 12 cm, so there was 3.5 cm of tissue and mesh overlap around the hernia orifice (Fig. 3).

The models were dynamically loaded by the impulse of air pressure. The load simulated an increased intra-abdominal pressure during different activities of a patient. Each model was loaded with different pressure: 14 kPa (corresponding to standing Valsava maneuver or walking on stairs), 18 kPa (jumping or coughing) and 25 kPa (jumping, vomiting)^22^. Load application was rapid. For the first load case the pressure grows from 0 to 14 kPa within 0.027 s, stays at maximum for 0.093 s and drops to 0 within 0.055 s. In the second model the 18 kPa pressure is reached within 0.048 s, stays at maximum for 0.052 s and drops to zero within 0.084 s. In the third model loading stage lasts for 0.054 s, the maximum of 25 kPa is kept for 0.041 s and drops to 0 within 0.083 s.

In each case the deflection of the mesh center was measured, and then used for numerical models validation. The main question, however, concerned the possible failure of the mesh-tissue junction under the given load.

### Numerical model of the implant fixed in the abdominal wall

The study was supplemented by numerical simulations of the Physiomesh implanted in the abdominal wall and subjected to intraabdominal pressure. The numerical model was defined by means of Finite Element Method (FEM) using Marc (Hexagon) commercial software. The idea of modelling of implanted surgical mesh was proposed by Lubowiecka^23^. In the present study, the implant is modelledd by a polygonal membrane structure supported in 12 points representing the joints of implant and the abdominal wall (staples). The elasticity of the abdominal wall is represented by elastic springs at joints—in the membrane plane and by elastic foundation around the hernia orifice^24^. To reduce the simulation time, only half of the implant was implemented using symmetry along the center of the PDO stripe. The material of the implant was modelled using a dense net model^23,24^. This type of modelling has been developed for technical fabrics and described in Ambroziak et al.^25^ Stiffness functions, determined for two directions in uni-axial tensile tests, were used this way to define the implant’s stiffness in two orthogonal directions like in a net of two types of threads. The finite element model was built using 4-node membrane elements with 3 translational degrees of freedom in each node.

The model was loaded respectively to the experimental conditions. This way one simulation referred to the load of 14 kPa and the second one to 25 kPa according to the experiment. Reaction forces calculated in the mesh supporting points, representing the forces in each joint, were calculated in both cases and compared to the experimentally assessed limit load of the tissue and implant connection.

### Uni-axial tests of the mesh samples

**Figure 2:**
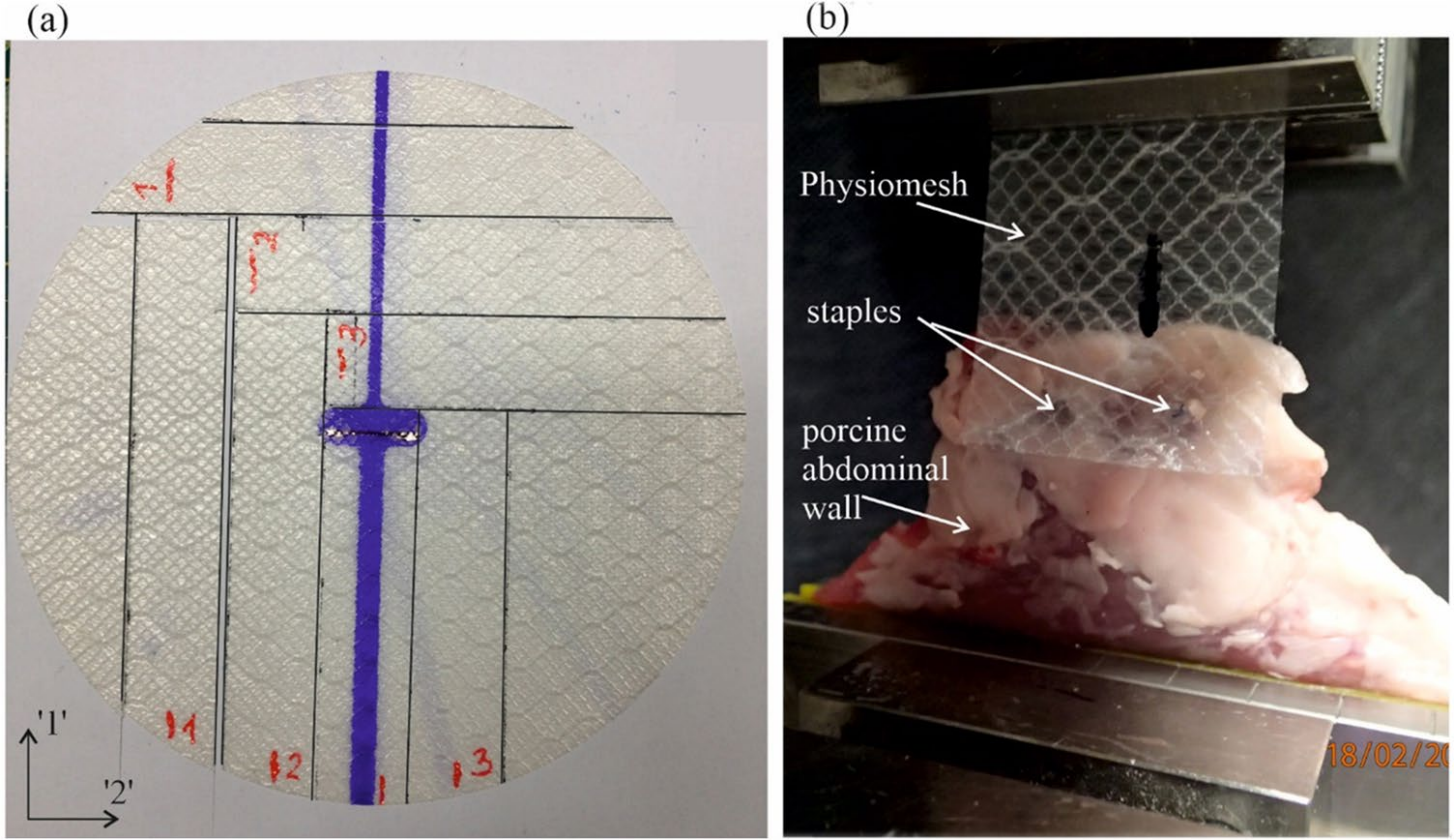
(**a**) Samples prepared for uni-axial tests. (**b**) Mesh-staples-tissue sample placed in the testing machine jaws.

Knowing that the largest stretch of the abdominal wall is in the cranio-caudal direction, it was reasonable to recognize the mesh mechanical properties in the direction of the violet stripe, which was recommended by the producer to be aligned with this direction. Axis of the stripe is named here as direction ‘1’. Moreover, the mesh reveals anisotropic properties which can be sensed manually—it stretches differently in different directions. Thus, the direction perpendicular to the violet stripe (direction ‘2’) was selected for the investigation as well. With this background, three pieces of two kinds of rectangular samples were cut from one piece of the implant (only one piece was available for the tests): with the longer side aligned to the violet stripe and perpendicularly to the stripe (Fig. 2a). Also, a sample containing the stripe was prepared. Each sample was 25 mm wide and minimum 110 mm long to have 90 mm clamp-to-clamp distance prior to the tests. Each sample represented the elementary pattern (contained the thicker and thinner threads). Zwick/Roell Z020 strength machine with video-extensometer was used. Static test, with constant strain rate equal to 0.001 1/s was set and the samples were stretched until rupture. The material parameters determined based on that will be used in dynamic calculations. Formerly, a comparative study was performed on the influence of strain rate in uni-axial tension test performed on other polypropylene mesh, DynaMesh-IPOM (FEG Textil-technik mbH, Aachen, Germany). No significant difference was observed between stress–strain relations obtained for strain rates 0.001 1/s and 0.03 1/s (dynamic test)^26^. On the other hand, there was a problem in the present study with samples fixation in the machine jaws. Grooved inserts to machine jaws (flat gripping system) have been used and there was a problem with the samples slipping of the fixation, due to slippery mesh coating. Curved gripping system, dedicated to membranes, could not be used in this study because of too small samples. Taking all this into account, standard static tests were performed.

The tension force and the samples elongations were recorded and the parameters of the mesh material law was identified based on that.

### Uniaxial tests of the mesh-staples-tissue system

Rectangular pieces of the mesh and porcine tissue with abdominal fascia on one side were prepared for testing. Two joints fixed the mesh in the fascia as presented in Fig. 2b. Three samples were prepared and stretched in Zwick/Roell Z020 machine until the mesh-tissue junction failure and the maximum load sustained prior to failure (limit load) was noted.

### Ethics approval and consent to participate

This research study was conducted retrospectively from data obtained for clinical purposes. All methods were performed with the relevant guidelines and regulations and approved by Ethics Committee by District Medical Chamber in Gdansk. No experimental protocol was included. All protocols were maintained according to law regulations in Poland. Informed written consent was obtain from all subjects included in study.

### Research involving animals

All methods including animal species were carried out in accordance with relevant guidelines and regulations in Poland, including ARRIVE guidelines. All methods were approved by Ethics Committee by District Medical Chamber in Gdansk.

### Previous communication

Results and conclusions of this study was presented during the oral session in European Hernia Society Congress in 2023 in Sitges.

## Results

### Ex-vivo model of mesh-staples-tissue under ‘intra-abdominal’ pressure

**Figure 3:**
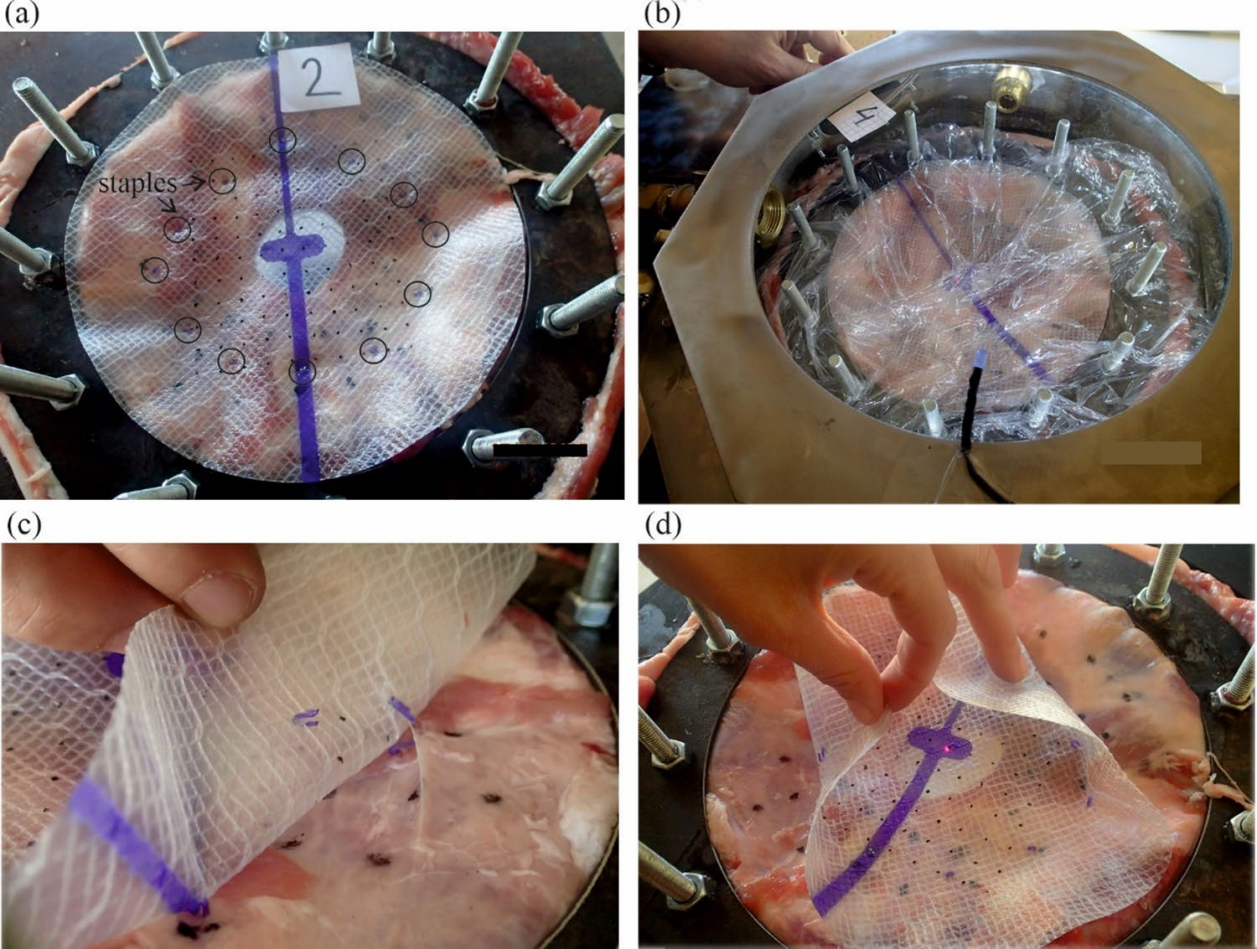
(**a**) operated hernia model, (**b**) model in the pressure chamber, (**c**) failure mode after pressure impulse of 18 kPa, (**d**) failure mode after pressure impulse of 25 kPa.

The first model was loaded with a pressure of 14 kPa. The load impulse was repeated five times and no damage was observed in the model. Then, an increased pressure strike was applied, with a value of 25 kPa and the model failed—two staples were pulled out of the tissue. The second model was loaded with a pressure of 18 kPa. This model failed after first impulse—one staple broke and one was pulled out of the tissue. The impulse pressure applied to the third model had the magnitude of 25 kPa and the model failed in the first load—one staple broke and two were pulled out of the tissue. The model and the failure modes after the pressures of 18 and 25 kPa are presented in Fig. 3c and Fig. 3b. In each case no plastic deformation was observed, which points at elastic behaviour of the mesh in the considered range of load.

In every failure mode the fixation damage occurred in the oblique direction in relation to the PDO stripe of the mesh, with the approximate angle of 45°. That suggests, that the highest force occurs in this direction. Taking into account the identified strength of the fixation in the Physiomesh/Securestrap system one can speculate, that with the impulse pressure of 14 kPa the junction forces in the mesh fixation points do not exceed 4 N. But for the pressures with the magnitudes higher than 18 kPa the reaction forces exceed 4 N. The results of the numerical simulations verified that hypothesis.

### Stiffness functions of Physiomesh and limit load of mesh-staples-tissue system

**Figure 4:**
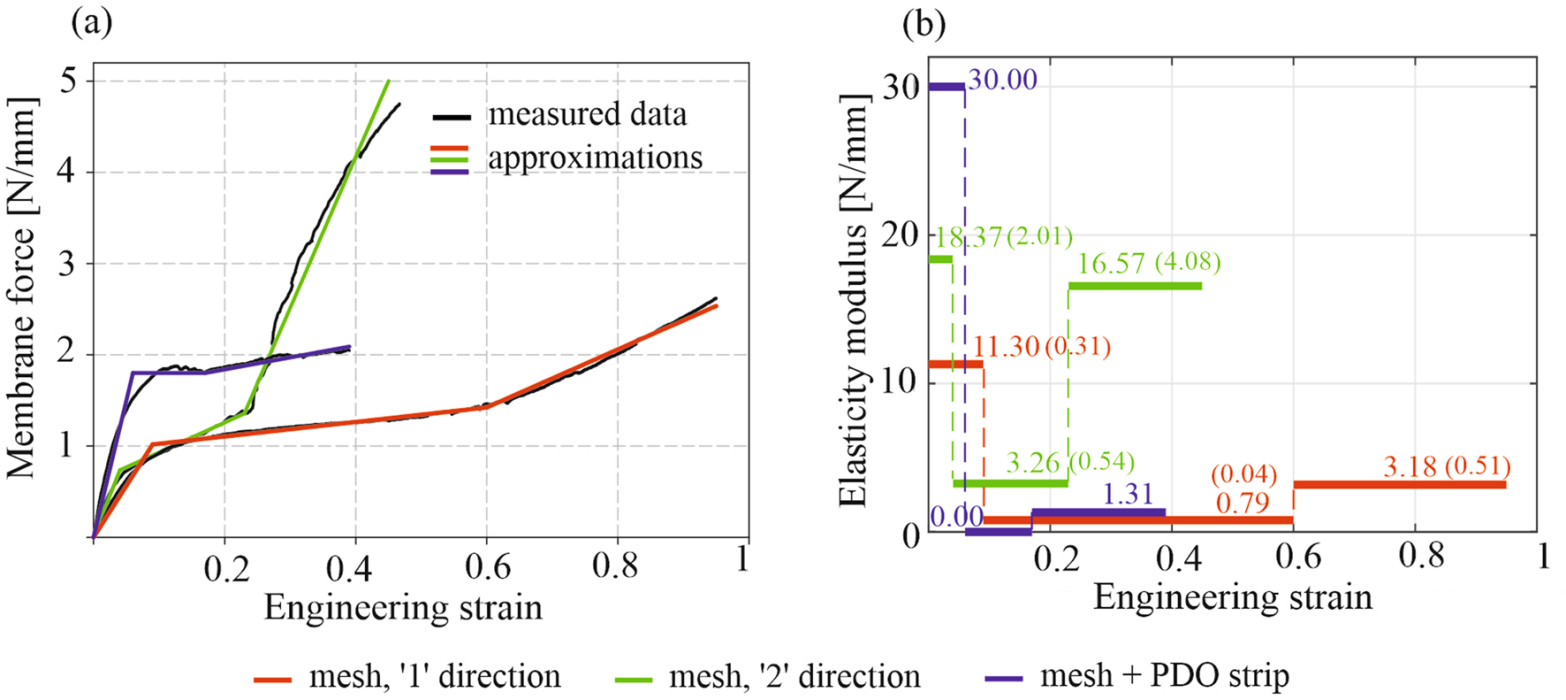
(**a**) Averaged membrane forces vs engineering strain measured for Physiomesh samples with three-linear approximations. (**b**) Elasticity moduli of the mesh in ‘1’ and ‘2’ directions and of the mesh + PDO stripe; standard deviations of the moduli are written in brackets.

The membrane forces (calculated as a tension force divided by the sample width) obtained in uni-axial tests vs engineering strains (calculated as a ratio of the sample elongation and its initial length) are presented in Fig. 4a (averaged results). The relations showed nonlinearity and anisotropy of the material and also its heterogeneity (different stiffness of the mesh covered by the PDO stripe than the non-covered part) of the mesh. These mechanical properties of the mesh were reflected in the numerical model by setting appropriate stiffness functions in the material definition. That stiffness was different for ‘1’ and ‘2’ directions of the mesh and also different in the area covered by the PDO stripe. Thus, although approximate, the dense net material model represents the mechanical behaviour of the implant including its changing stiffness, as shown in Fig. 4, under increasing tensile stress. As the laboratory dynamic tests of operated hernia models showed elastic behaviour of the mesh in the range of the considered pressure load, elastic stiffness functions were selected for modelling the mesh behaviour in numerical model. To approximate the material nonlinearity, three-linear approximations of the stress–strain relations were made by means of Marquardt–Levenberg variant of the least squares method and the stiffness functions were determined by tangent modulus of elasticity identified for different strain ranges^26^. The results are presented in Fig. 4b. The higher the elasticity modulus value the higher the stiffness of the implant in a considered direction. A stiffness decrease is visible for each kind of sample after presenting some initial stiffness. That occurred due to material delamination during tension—the coating separated from the knitted structure. When this finished, the mesh core started individual work, presenting some stiffness increase. The effect of the mesh stiffening recovery is at most visible for the samples cut along ‘2’ direction of the material. Description of this complex behaviour of the material is out of scope of the present study as no delamination is observed in the laboratory model of operated hernia. Elastic material model is described here in a full range of the uni-axial test, however it can be applied only to model first load path of the mesh, as the mesh behaviour is not reversible when its delamination begins.

The initial stiffness of the mesh is the most important, because that property is valid at the beginning of the mesh load (for the initial strain range). One can notice the highest initial stiffness of the PDO stripe, intermediate value for the mesh in ‘2’ direction, while initial stiffness is the smallest in the ‘1’ direction (Fig. 4b). However, in the initial range of the mesh strains, its mechanical property in the ‘1’ direction is dominated by the PDO stripe, which is parallel to ‘1’ direction and which stiffness was almost threefold higher that the stiffness of the mesh alone in the ‘1’ direction. For strains higher than approximately 0.1 the stiffness of the stripe strongly decreased. Limit load of the mesh-tissue junction, in a single junction point, was identified as 4 N, which is average result of three tests. Each time the failure was due to pulling the staple out of the tissue.

### Results of numerical simulations

**Figure 5:**
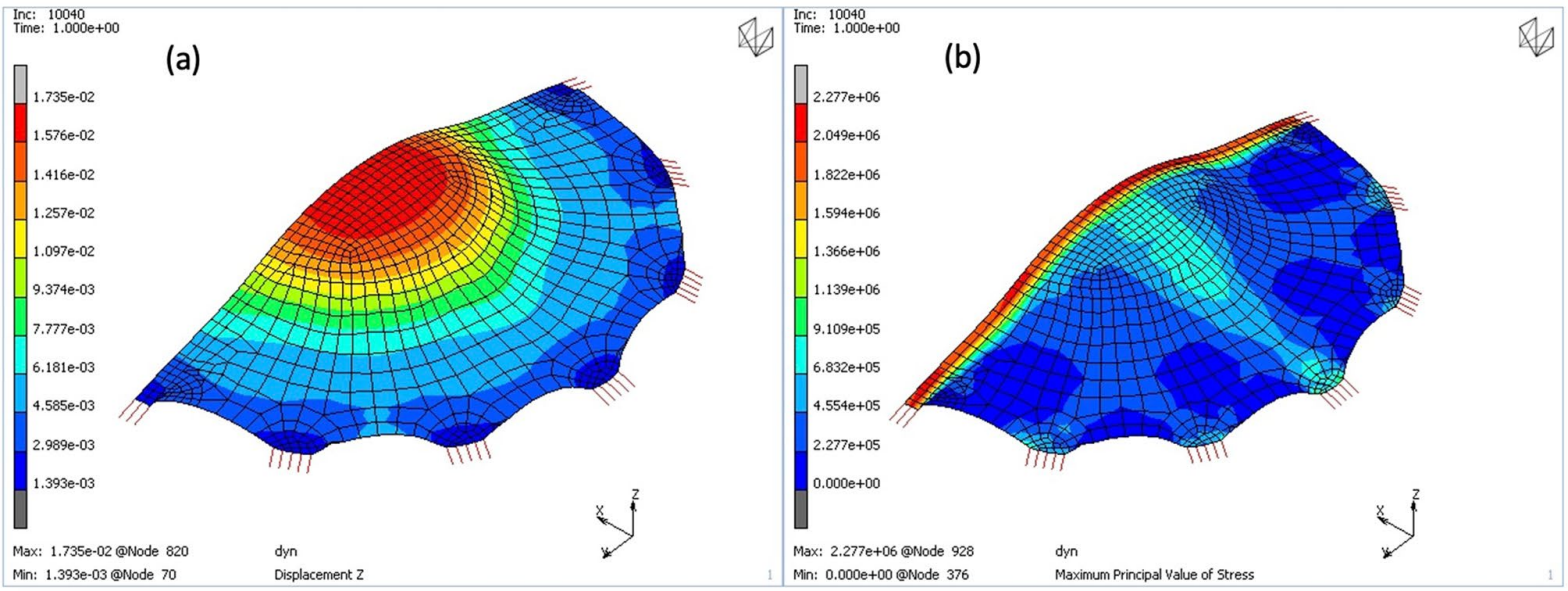
Displacement of the Implant model under pressure of 14 kPa (**a**) and principal stress (**b**).

The reaction forces of the model that represent the forces acting on a staples were calculated in two load scenarios, with pressure value of 14 kPa and of 25 kPa, referring to the experiment. In those two cases the maximum forces obtained were 3.79 N and 5.29 N respectively. That means that if applying the higher pressure, the forces in joints exceed their strength identified experimentally. The maximum displacements (while the mesh bulges under pressure) in both simulations were 17 mm and 23 mm respectively to the loading conditions (Fig. 5a).

A stress concentration can be easily noticed in the PDO stripe (Fig. 5b). This is caused by the higher stiffness of the stripe (built-in to the implant) comparing to the rest of the membrane.

## Discussion

The aim of this study was to investigate the mechanical behavior of the Physiomesh/Securestrap system, which experienced high failure rates and adverse clinical outcomes. The authors conducted a set of mechanical experiments and numerical simulations to gain insight into the potential cause of system failures and understand the mechanics of the implant fixed to the anterior abdominal wall.

Figure 1b shows geometry of the Physiomesh/Securestrap. It can be noticed, the staple is so narrow that it can easily miss any thread of the mesh while being applied. Then, the staple fixes absorbable barrier layers only to abdominal tissue, which gives no fixation of the mesh because this layer hardly has any load bearing capacity and is damaged by the staple due to any load acting on the implanted mesh (intraabdominal pressure or body movement). This incompatibility of the sizes of the staple and the mesh pores is a first, basic drawback of the Physiomesh/Securestrap system. When move to the mechanical analysis of the system, subsequent drawbacks are found.

The elasticity modulus of the Physiomesh was determined through uniaxial tension tests, showing anisotropy and non-homogeneity of the material. These properties were considered in the numerical model, which accurately represented the behavior of the implant. However, the analysis of the uni-axial tests results revealed a second major drawback of the mesh, which is the idea of using PDO stripe as an orientation marker. The values of the elasticity modulus in the initial tension state identified in ‘1’ and ‘2’ directions of the mesh and also for the PDO stripe show that this stripe is approximately 3-times stiffer than the mesh itself in the ‘1’ direction (parallel to the stripe). The stripe changes a basic demanded property of the mesh, which states the mesh stiffness should be smaller in the cranio-caudal direction (after implantation) than in the lateral direction. That property would be preserved for Physiomesh without the stripe. As Fig. 4a also shows, the stripe has the lowest limit load and limit strain among other tested mesh samples. Physiomesh implanted according to the manufacturer recommendation causes a mismatch between the mesh properties and natural kinematic properties of the abdominal wall, which induce increased reaction forces in the mesh fixation points and causes the failure^11^. Former studies show the mesh orientation importance on the reaction forces in the mesh fixation points^27^.

The uni-axial tests of the mesh-staples-tissue system provided valuable information about the strength of the mesh-tissue junction. The results indicated that the limit load of the junction was identified as 4 N, with failure primarily occurring due to the staple pull-out from the tissue. This finding suggests that the fixation strength of the Physiomesh/Securestrap system may not be sufficient, especially under higher loads. As a comparison, we identified the junction capacity of Ventralight/Sorbafix system experimentally in uni-axial tensile test according to the testing protocol described above. The limit load of the Ventralight/SorbaFix system is 9.7N (average value from test on six samples) meaning the capacity of this system is greater than Physiomesh/Securestrap. This result can be one pieces of evidence standing for the Ventralight/SorbaFix system as a solution better than Physiomesh/SecureStraps.

The ex-vivo experiments on the porcine abdominal wall models implanted with the Physiomesh/Securestrap system under simulated intra-abdominal pressure load provided insights into the mechanical behavior of the system. The models exhibited failure, such as staple breakage or pull-out, particularly under higher pressure loads. The failure modes consistently occurred in an oblique direction relative to the PDO stripe of the mesh. However, the highest calculated junction force value is observed in the joint on the PDO stripe. One should be aware of the fact that the material model of implant identified in uni-axial test does not fully describe the multi-axial behaviour of knitted surgical mesh. In particular, since the specimens were cut correspondingly to the indications regarding the direction of mesh implantation (along the PDO and perpendicular to its axis), which does not necessarily correspond to the structure of the implant.

The numerical simulations further supported the experimental findings, demonstrating reaction forces on the staples and maximum displacements under different pressure scenarios. The results indicated that the forces in the fixation points exceeded their experimentally determined strength, particularly under higher pressure loads.

The numerical simulations showed how the PDO stripe itself seems to influenced negatively the implanted mesh behaviour, causing a stress concentration along this stiffer part of the material under pressure (Fig. 5b). It may be important in particular, when it is oriented along a direction of the largest abdominal strains generated in patient’s postoperative life.

The mesh exhibited high flexibility in transverse projection – probably intended to compensate for rotation movements of the torso. In turn, in the longitudinal projection the flexibility was insufficient. The reason for that is the strengthening of the mesh with the extra stripe which influenced negatively overall behaviour of the implanted mesh.

On the other hand there are assumptions and hypotheses encountered in the literature which try to explain the mechanism of recurrence cases of Physiomesh/Securestrap system usage. One, partially true, assumes the lack of strength of the non-absorbable implant layer to the breaking forces generated by the maximum intra-abdominal pressure^22^. This is also confirmed by experimental research conducted by our team. There are not enough strong threads within the knitting pattern, which directly contributed to breakage of the implant from the fixation system. To be strict, the mesh is knitted out of threads of two diameters (see Fig. 1b). Our preliminary study showed that when staple embraces thinner thread, the thread can be ruptured while the system is loaded by pressure. Thus, in our tests we placed staples always around the thicker threads to simulate best surgical situation of the considered system. Besides, the staple itself was made without proper consideration of the tissue specificity. It was brittle and broke several times when placed in the physical model.

These findings shed light on the potential biomechanical factors contributing to the high failure rates observed with the Physiomesh/Securestrap system. The inadequate fixation strength, demonstrated by the staple pull-out and breakage, suggests a mechanical limitation of the system. The stress concentration observed in the PDO stripe highlights the importance of considering the mechanical properties and design of the implant when assessing its performance. Those factors also recur in other biomechanical studies^28–30^.

Physiomesh implant with the Securestrap fixation system was approved for use in the USA by the Food and Drug Administration (FDA) in 2010. In its application, the manufacturer (Ethicon) declared that it is essentially similar to three other implants of this company, which have been approved for use before. Very quickly, the FDA began receiving information about a disturbingly high complication rate after Physiomesh/Securestrap system use. Physiomesh was permanently withdrawn from the market in May 2016 and as of today, the number of court claims has reached nearly 3000^13^. The whole situation is worth considering, as the concept of the new system seemed to coincide with the commonly used.

The implications of this study extend beyond the specific case of the Physiomesh/Securestrap system. By understanding the mechanical behavior of implant-tissue systems, researchers and clinicians can make informed decisions regarding implant selection, design modifications, and fixation techniques to improve surgical outcomes. This knowledge may guide the development of future generations of implants, with a focus on creating mechanically reliable systems compatible with the mechanics of a healthy abdominal wall^28,29,31^.

The analysis described above was partially inspired by the discussions with patients who experienced hernia recurrence after surgery with a Physiomesh/Securestrap system. The patients were often able to accurately describe the moment of recurrence. The recurrence most often appeared in a moment of and abrupt sneeze, cough or sudden twisting of the torso accompanied by severe pain and/or feeling of “tearing tissue”. In our model we took into account one of the situations, generating a high intraabdominal pressure, and explain the biophysical reasons for this state of affairs.

Based on experimental studies on an animal model (Majercik et al., Iannitti et al.), we know that the first two weeks are crucial for maintaining position in the case of defects repaired using the IPOM technique^32,33^. This is a period when there is no incorporation of the mesh, and the implant itself is held in its position solely by the force of the mesh-fascia-staple junction. In the case of the Physiomesh/Securestrap system, we believe that most of such a high percentage of recurrences (compared to other implants) occurred within the first few days after the procedure. Patients described this as a sudden pain after performing activities that increased intra-abdominal pressure (cough, bending, etc.). However, the moment when they reported to the doctor was delayed by several months—this was due to the fact that the patient began to realize the recurrence only when he noticed a protrusion on the previously torn implant^15^. In the past, our team has conducted repetitive stress tests on individual implants to replicate as closely as possible the conditions prevailing, for example, during a coughing fit, persistent vomiting, etc.^21^. However, in the case of Physiomesh/Securestrap, this was not necessary. The mesh fixation broke under physiological pressure (apart from 14 kPa) in single load tests that we did not study mechanics under repetitive load.”

Without maintaining mechanical stability, the initiation of the incorporation process is impossible. We derive our knowledge on this from, among others, the works of Kallinowski et al. focusing on the GRIP CONCEPT, that is, critical resistance to impacts^28,31,34^.

In preclinical studies on the porcine and rabbit model, Ethicon presented great results in terms of both fixation, maintenance in the operating field, and tissue incorporation^35,36^. The presented analysis proves that the animal model cannot be directly translated into humans. The reasons include both phylogenesis with the adoption of a vertical body posture by humans, as well as the anatomy of the anterior abdominal wall, and thus a different distribution of forces and directions of their action in the event of a sudden increase of intraabdominal pressure than in the case of four-legged animals.

Comparing with a number of studies referring to biological aspects of mesh implantation based on an animal model, the above results also allow to understand the biomechanical cause of such rapid and spectacular hernia recurrences in patients operated on with the Physiomesh implant. We believe that this will prevent similar errors in the future, while also serving as a valuable reference for the development of new implants^17^. Additionally, it shows the complexity and importance of this mechanism in terms of the mesh-fascia system on the level of physics.

It is important to note that this study has certain limitations. Uni-axial tests of the mesh and fixation system have been made for limited number of samples. However, the results considered as average confirm the mesh anisotropy and significant mesh stiffening in the area covered by PDO stripe. That features could be sensed manually for this mesh. On the other hand, nowadays bi-axial tests are considered more suitable than uni-axial to identify parameters of constitutive law of material working in a complex stress state. Thus, in the future study such test should be performed in a case of abdominal mesh. Also, dense net model for implant material does not considers either yarns interactions or the textile coating. Moreover, the cut of specimens of the mesh prepared for the material identification corresponded to the indications regarding the direction of mesh implantation (along the PDO and perpendicular to its axis), which did not necessarily correspond to the structure of the implant.

The experiments and simulations were conducted under controlled laboratory conditions, which may not fully replicate the complex in vivo environment. There was porcine tissue used in an ex vivo experiment that may lead to slightly different results comparing to in vivo human tissue behaviour. Additionally, the study focused on mechanical aspects and did not consider other factors that may contribute to the clinical failures observed with the Physiomesh/Securestrap system, such as biological responses and host reactions.

What is interesting, when Physiomesh/Securestrap entered the market in 2013, surgical meshes were classified as category II according to the regulations of the European Union and the USA. This meant that the introduction of a new product for general use required no clinical trials. From October 2021, upon entry into force of the MDR (Medical Device Regulation), hernia meshes have been included in group III, which means that randomized control tests must be carried out before placing the implant on the market^16^.

We firmly believe that the presented findings will prevent similar mistakes in the future, but at the same time will allow us to supplement our current knowledge in the field of the anterior abdominal wall mechanics.

## Conclusions

In conclusion, this study provides a critical insights into the mechanical behavior of the Physiomesh/Securestrap system, highlighting the need for better understanding of the biomechanical factors involved in abdominal hernia repair. By considering these factors, future advancements in implant design and fixation techniques may be made, ultimately leading to better surgical outcomes and reduced complications for patients undergoing abdominal hernia repair surgeries. Hopefully, results allow avoiding similar errors in the future. The findings not only expose the cause for the failure of Physiomesh/Securestrap system, but also allow better understanding of the forces acting within the abdominal cavity, as well as to broaden our knowledge in this field. Optimally, this will prevent similar situations from arising in the future.

## Data Availability

The datasets used and/or analysed during the current study available from the corresponding author on reasonable request.
